# The Decadence of the Negro

**Published:** 1897-04-24

**Authors:** 


### The Decadence of the Negro.

Those who have grown old in the Southern States of America declare that the negro is not so hardy as he was in the days of slavery. Dr. Walton, of San Antonio, has lately collected some statistics on this subject which are of considerable interest, as at least establishing the fact that the negro in the Southern States, notwithstanding the intellectual progress that he has made during his quarter of a century of freedom, dies in far larger numbers than the whites living in the same districts. Especially does this apply to the large cities, where the death-rate among the coloured people is nearly double that of the whites. It is asserted by some that in the old days consumption was quite an infrequent cause of death among the negroes. Now, however, that disease is considerably more frequent among them than among the whites. If we look for the causes of this deterioration probably the first is the greater struggle for existence. Poverty, which is the lot of so large a proportion of the coloured population, means deprivation and overcrowding with all its resulting evils. In the days of slavery, although he had no luxury, the negro was reasonably comfortable, and his surroundings and mode of life were more healthy than they often are now. He lived in the country, whereas he now crowds into the towns; his log cabin let in more air than the houses in which he now often dwells; he was fairly fed, he kept better hours than he does now, and he was debarred from many excesses in which he indulges now without stint. Perhaps there are other causes still more subtle. The negro is no longer a pure African. The race is fast losing its individuality—it is a multi-coloured, instead of a black race—and it would seem that this gradual admixture of Caucasian blood has done good to neither party, having resulted in a race anything but robust. At any rate, we are told that the majority of the negroes who die of consumption are of the lighter element. But here, again, it is by no means certain that other causes are not in operation besides racial instability. However completely we may accept the statistical facts presented to us, it is still clear that the explanation of them is a complex matter, and that they are open to various interpretations.

